# Toxoplasmosis in patients with an autoimmune disease and immunosuppressive agents: A multicenter study and literature review

**DOI:** 10.1371/journal.pntd.0010691

**Published:** 2022-08-08

**Authors:** Marie-Fleur Durieux, Jean-Guillaume Lopez, Maher Banjari, Karine Passebosc-Faure, Marie-Pierre Brenier-Pinchart, Luc Paris, Gilles Gargala, Sabine Berthier, Julie Bonhomme, Cathy Chemla, Isabelle Villena, Pierre Flori, Emilie Fréalle, Coralie L’Ollivier, Florian Lussac-Sorton, José Gilberto Montoya, Estelle Cateau, Christelle Pomares, Loïc Simon, Dorothée Quinio, Florence Robert-Gangneux, Hélène Yera, Marc Labriffe, Anne-Laure Fauchais, Marie-Laure Dardé

**Affiliations:** 1 Department of parasitology and mycology, Dupuytren University Hospital, National Reference Center of Toxoplasmosis, Limoges Cedex, France; 2 Department of internal medicine, Dupuytren University Hospital, Limoges Cedex, France; 3 Department of internal medicine faculty of medicine -Rabigh Campus- King Abdulaziz University, Jeddah, Saudi Arabia; 4 University Grenoble Alpes, CHU Grenoble Alpes, Parasitology-Mycology laboratory, Grenoble, France; 5 Parasitology laboratory, AP-HP Pitié-Salpêtrière, Paris, France; 6 Parasitology laboratory, University hospital of Rouen, Rouen, France; 7 Department of internal medicine, University hospital of Dijon, Dijon, France; 8 Microbiology laboratory, University hospital of Caen, Caen, France; 9 Parasitology Laboratory, EA 7510, Reims Champagne Ardenne University, National Reference Centre on Toxoplasmosis CHU Reims, Reims, France; 10 Parasitology laboratory, Hospital of Saint-Étienne, Saint-Étienne, France; 11 Parasitology laboratory, University hospital of Lille, Lille, France; 12 Aix Marseille Univ, IRD, AP-HM, SSA, Vitrome, Marseille, France; 13 Parasitology laboratory, University hospital of Bordeaux, Bordeaux, France; 14 Dr. Jack S. Remington Laboratory for Specialty Diagnostics, Palo Alto, California, United States of America; 15 Parasitology laboratory, University hospital of Poitiers, Poitiers, France; 16 Parasitology-Mycology laboratory, Côte d’Azur University, INSERM 1065, University hospital of Nice, Nice, France; 17 Parasitology laboratory, University hospital of Brest, Brest, France; 18 Parasitology laboratory, Univ Rennes, CHU Rennes, France; 19 Parasitology laboratory, AP-HP Cochin, Paris, France; 20 Pharmacology & Transplantation, INSERM U1248, Université de Limoges, Limoges, France; George Washington University, UNITED STATES

## Abstract

**Background:**

Cases of *Toxoplasma* reactivation or more severe primary infection have been reported in patients receiving immunosuppressive (IS) treatment for autoimmune diseases (AID). The purpose of this study was to describe features of toxoplasmosis occurring in patients with AID treated by IS therapy, excluded HIV-positive and transplant patients.

**Methods:**

A multicenter descriptive study was conducted using data from the French National Reference Center for Toxoplasmosis (NRCT) that received DNA extracts or strains isolated from patients, associated with clinical data. Other cases were retrieved through a questionnaire sent to all French parasitology and internal medicine departments. Furthermore, a systematic literature review was conducted.

**Results:**

61 cases were collected: 25 retrieved by the NRCT and by a call for observations and 36 from a literature review. Half of the cases were attributed to reactivation (50.9%), and most of cases (49.2%) were cerebral toxoplasmosis. The most common associated AID were rheumatoid arthritis (28%) and most frequent treatments were antimetabolites (44.3%). Corticosteroids were involved in 60.7% of cases. Patients had a favorable outcome (50.8%) but nine did not survive. For 12 cases, a successful *Toxoplasma* strain characterization suggested the possible role of this parasitic factor in ocular cases.

**Conclusion:**

Although this remains a rare condition, clinicians should be aware for the management of patients and for the choice of IS treatment.

## Author summary

Severe forms of toxoplasmosis are well described in HIV-infected or transplant patients. However, they have been described in a less studied category of patients: those with autoimmune diseases and treated with immunosuppressive agents, corticosteroid and biotherapies. We report, conjointly with the French National Reference Center for Toxoplasmosis, the largest collection of cases of toxoplasmosis occurring in these patients to clarify the risk factors and epidemiology associated with these serious forms of toxoplasmosis. In our series, as well as in the literature, rheumatoid arthritis and systemic lupus erythematosus are the most common autoimmune diseases associated with toxoplasmosis, and antimetabolites, anti-TNF-α and corticosteroids were involved in most cases. The originality of this work lies in the molecular characterization of *Toxoplasma* to study the possible role of the strain factor in diverse clinical forms. Atypical strains, as those observed in South America, were more often observed in ocular toxoplasmosis cases. The significant proportion of cerebral toxoplasmosis (49,2%) and a 14.8% mortality risk highlight the necessity to inform clinicians of this opportunistic infectious complication and encourages them to evoke toxoplasmosis quickly even in case of occurrence of non-specific symptoms.

## Background

*Toxoplasma gondii* infects a large part of the world’s population. After a primary infection, tissue cysts containing thousands of latent *T*. *gondii* bradyzoites persist lifelong in different organs, mainly brain. An immunocompetent host may experience a self-limiting febrile illness, but in the immunocompromised, this infection can be devastating. Profound immunosuppression in solid organ or stem cell transplant and advanced human immunodeficiency virus (HIV) disease is well-known to predispose patients to severe forms of toxoplasmosis [1]. However, cases have also been reported in patients with other types of immunosuppression: those who take immunosuppressive (IS) treatment for inflammatory disorders and autoimmune disease (AID) [2]. These clinical presentations may result from a primary infection or reactivation of a latent infection, and risk factors concerning these situations are poorly understood.

This is of potential concern given the increase in immunosuppressive drugs for treatment of inflammatory conditions. The emergence of various biologic therapies has brought a great clinical benefit for patients with AID, but severe opportunistic infections more frequently arise. Toxoplasmosis is a part of it, but this risk remains difficult to grasp because it is usually multifactorial and the specific risk factors are not yet known [2]. To our knowledge, there is little data on opportunistic toxoplasmosis prevalence or incidence in patients with AID. Redondo-Benito *et al*. in a retrospective study (1986–2014) of patients with idiopathic inflammatory myopathies found a 6.4% prevalence of opportunistic infections, with only two cases of toxoplasma retinitis among 204 patients [3]. Few other reviews are available in the literature describing toxoplasmosis occurring in non-HIV immunosuppressed patients, and data are often not sufficient and contradictory to draw robust conclusions on rates of opportunistic infections. Moreover, the utility of *Toxoplasma* prophylaxis in patients taking immunosuppressive drugs is unclear, with no validated strategy to identify high-risk patients or to guide the choice and duration of prophylactic drugs.

The complexity of the problem lies in the diversity of action of these immunosuppressive molecules. It is evident that these treatments target key molecules involved in the host defense against *T*. *gondii*. The basis of the susceptibility of these patients to toxoplasmosis is the imbalance induced by immunosuppressive drugs in the subtle host-parasite equilibrium that usually leads to the establishment and maintenance of a chronic infection. During the acute stage of *T*. *gondii* infection, TNF-α, a cytokine with pro-inflammatory and anti-infectious properties by stimulating the phagocytic activity of leukocytes, is involved in host cellular immune response. Interleukin-12 (IL-12) produced by macrophages, neutrophils, monocytes and dendritic cells plays a key role in the innate immunity against *T*. *gondii* [4]. CD8^+^ T cells, activated by IFN-γ and IL-12, are major effector cells of the immune adaptive response in the control of infection, however some strains of *T*. *gondii* may inhibit the production of IL-12 by macrophages and then limit the production of inflammatory cytokines [5]. One recent IS molecule, ustekinumab, has an action on this pathway by inhibiting IL-12. Rituximab, targeting the protein CD20 (a B-cell specific marker), leads to a decrease in inflammatory cytokines, including IFN-γ and IL-12 [6]. Methotrexate, one of the most frequently used treatment in AIDs, has multiple targets, affecting both the cellular and humoral components of the immune system and inhibiting pro-inflammatory cytokine production [7]. Azathioprine and mycophenolate mofetil (MMF) use complex mechanisms of actions, but both inhibit the synthesis of deoxyribonucleic acid (DNA) bases. In addition, corticosteroids are also very frequently given in these diseases. They have both anti-inflammatory and immunosuppressive activities, acting on multiple molecular targets. They increase infectious risks and alter the symptomatology of infections. Corticosteroids are often used in combination with other IS treatments, strengthening the deleterious effects. In addition to all these pharmacological classes, many new molecules have appeared in recent years, the long-term effects of which are not yet well known.

The purpose of this study was to describe clinical and biological features of toxoplasmosis occurring in HIV-negative patients in France, who had an AID and had been treated with IS therapies, including biotherapies. Our data, obtained through the French National Reference Center for Toxoplasmosis (NRCT) and via a questionnaire sent to internal medicine departments, was completed by a systematic literature review.

## Material and methods

### Ethics statement

Anonymized clinical and epidemiological information associated to the *Toxoplasma* strains collected at the French NRCT are reported *via* internet data entry by the 37 specialized laboratories (through http://cnrtoxoplasmose.chu-reims.fr) using a specifically developed software, ToxoSurv (EpiConcept). This software and the data collection were approved by the French National Data Protection, the “Commission Nationale de l’Informatique et des Libertés” (CNIL approval reference DR-2016-186). A letter for informed consent explaining the nature and possible consequences of the studies is provided via the French National Reference Center for Toxoplasmosis.

### Current study

**Fig 1:**
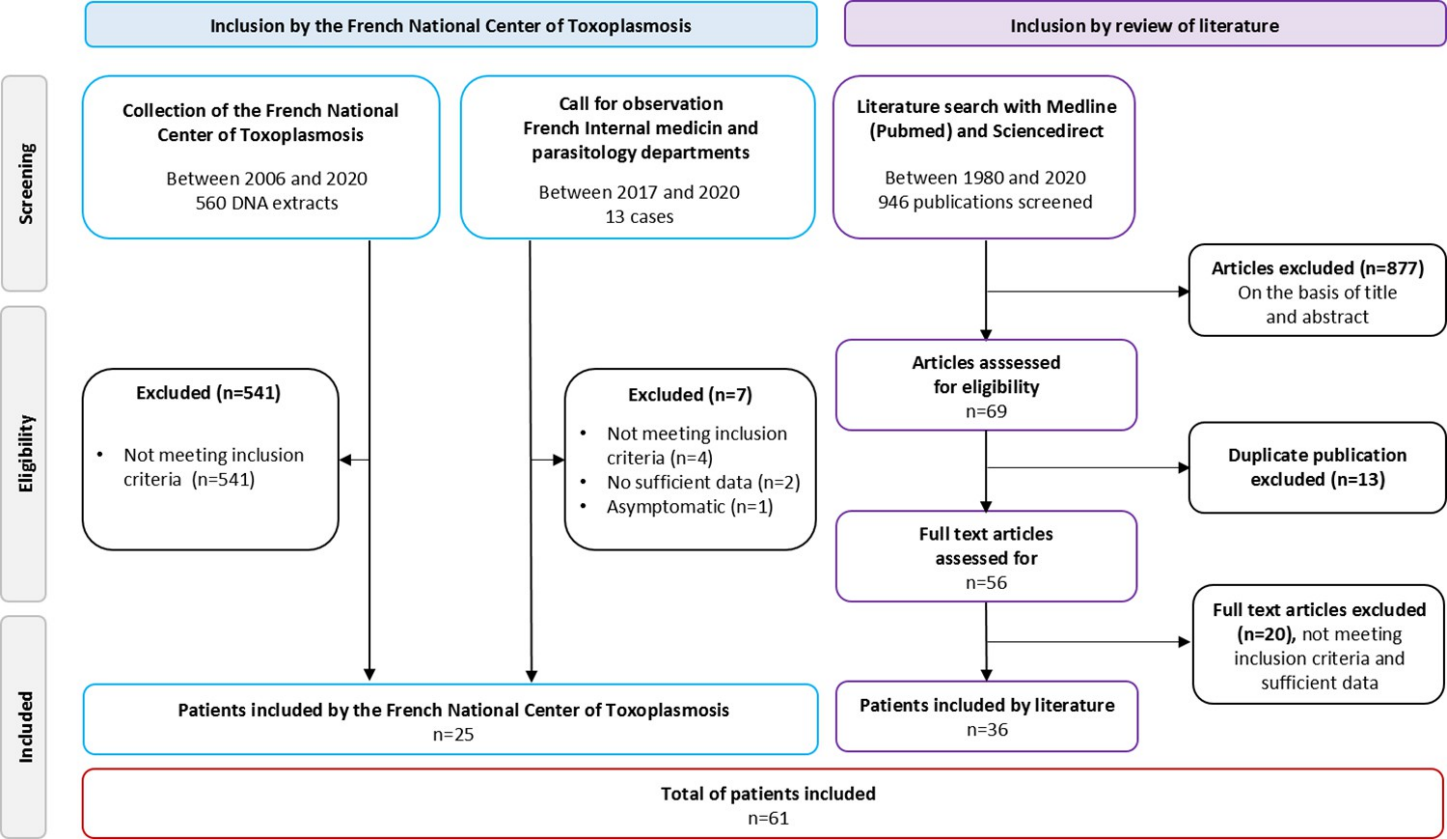
Flow diagram of patient selection process.

First source of recruitment was cases recorded via the NRCT associated laboratory in charge of *Toxoplasma* strain typing (Limoges, France) from 2006 to 2020. This laboratory received *Toxoplasma* DNA extracts or *Toxoplasma* strains isolated from diverse forms of toxoplasmosis in parasitology departments from all over France. This case report is done by NRCT correspondents on a voluntary basis and allows collection of associated epidemiological and clinical data on a specific website. To complete this NRCT database, a call for observation was launched to the network of the 37 parasitology departments participating to the NRCT, and to all internal medicine departments across the country. A questionnaire collecting more detailed data (underlying diseases, clinical and biological data, medications for toxoplasmosis, administration of IS treatment, outcome) was sent to each physician in charge of the cases. Our inclusion strategy is summarized in Fig 1.

Patients with corticosteroids and/or IS drugs and diagnosis of toxoplasmosis during treatment or within months of discontinuation of their treatment were included. Our study excluded HIV-positive, transplant (solid organs and bone marrow), chemotherapy-treated, neoplasm-affected patients, and patients with congenital immune deficiencies. Each toxoplasmosis case was classified as definite (presence of parasite in tissue or body fluids demonstrated by culture, PCR, cytology or histology) or probable (clinical and radiological evidence associated with a positive serology and response to treatment). Primary infection was defined by seroconversion of IgG or at least a two-fold increase of IgG titers associated with a low-avidity IgG and the presence of anti-*Toxoplasma* IgM antibody. Reactivation of a chronic infection may be associated with an increase of IgG titers with or without elevation or apparition of specific IgM or IgA. Cases were also classified depending on the localization of the toxoplasmosis: cerebral, ocular or disseminated if at least two different organs or organ and blood were affected. Asymptomatic forms of primary infection were excluded as well as pauci-symptomatic forms, not specific to immunocompromised patients, that often go unnoticed and thus not reported to the NRCT. Only unusual clinical presentations or positive PCR in body fluids are included.

### Genetic analysis of *Toxoplasma* strains

When available, *Toxoplasma* DNA extracts or *Toxoplasma* strains were genotyped at the NRCT laboratory. Genotyping was performed through amplification of 15 microsatellite markers distributed on 11 of the 14 chromosomes of the parasite in a multiplex PCR assay [8]. Statistical tools of genetic analysis applied to the results allow strain classification into the different groups already described in *Toxoplasma* literature [9].

### Literature review

A literature search for English resources from PubMed and Sciencedirect databases by two authors independently (MFD and JGL) was performed in 2020, for screening of clinical cases published between 1980 and December 2020. All case reports of toxoplasmosis occurring in patients who have an AID and have been treated with IS therapy including biologics were collected, using the keywords toxoplasmosis and diverse AIDs (Rheumatoid arthritis (RA), cutaneous vasculitis, necrotizing vasculitis, inflammatory bowel disease (IBD), inflammatory myopathies, multiple sclerosis, myasthenia gravis, pemphigus vulgaris, psoriasis vulgaris, sarcoidosis, systemic lupus erythematosus (SLE)), and a wide variety of IS drugs such as methotrexate, rituximab, ustekinumab, anakinra, certolizumab pegol, tocilizumab, TNF-α antagonists (etanercept, adalimumab, infliximab, golimumab), or other IS agents (MMF, azathioprine, cyclophosphamide, leflunomide, fingolimod, abatacept, trametinib, sirolimus). Conjunction words “and”/“or” were used to specify the search. In addition, the retrieved article reference lists have been manually searched for other potentially eligible studies.

The same researchers read titles and abstracts to select potentially eligible studies and according to the inclusion and exclusion criteria (duplicate case reports, commentaries, editorials, fundamental research, investigations with incomplete or missing main data, published in a language other than English, studies on animals). The detailed search strategy is presented in Fig 1. Afterwards, the full texts were read and data were collected on a database. Data extraction from the selected studies included: study identification (first author, publication date, journal publication); demographic data (age, sex); data concerning the AID, clinical symptoms, radiographic findings, treatment, and prognosis. Data were input and then checked by one of the authors responsible for analysis of the data. Any disagreement on the inclusion of a case was resolved through discussion with an additional author (MLD). After selection, 36 articles were eligible and were included in the study.

### Statistical analysis

P values were calculated with the use of two nonparametric tests: the Fisher’s exact test for discrete variables and the Mann-Whitney test for continuous variables. P value <0,05 was considered as statistically significant.

## Results

### Current study

From 2006 to 2020, the French NRCT laboratory received 560 samples originating from immunocompromised patients who developed toxoplasmosis. Among these, 19 corresponded with the inclusion criteria. The call for observation allowed to complete the list with six other cases. Finally, 25 cases were included, reported from 17 different centers in France, and from one U.S. laboratory (Tables 1 and 2 and S1 Data [10]). The most frequently concerned underlying AIDs were RA (20%) and SLE (20%), IBD (16%) and psoriatic arthritis (8%). Corticosteroids (64%), methotrexate (20%) and MMF (20%) represented the main IS treatments. In more than half of the cases (52%), therapy relied on an association of corticosteroids and IS treatment. In two cases, molecules used were not specified and for one patient suffering from systemic sclerosis, treatment with cyclophosphamide had been stopped a few months before the episode of toxoplasmosis.

**Table 1:** Underlying conditions and treatment of patients.

Presentation of patients	Current study	Literature review	Total
	N = 25	N = 36	N = 61
**Underlying condition N (%)**			
Rheumatoid arthritis	5 (20)	12 (33.3)	**17 (28)**
Systemic lupus erythematosus	5 (20)	9 (25)	**14 (23)**
Inflammatory bowel disease	4 (16)	3 (8.3)	**7 (11.6)**
Psoriatic arthritis	2 (8)	1 (2.8)	**3 (5)**
Multiple sclerosis	0 (0)	2 (5.6)	**2 (3.3)**
Sarcoidosis	1 (4)	1 (2.8)	**2 (3.3)**
Cryoglobulinemia	1 (4)	1 (2,8)	**2 (3.3)**
Ankylosing spondylitis	0 (0)	1 (2.8)	**1 (1.6)**
Connective tissue disease	0 (0)	1 (2.8)	**1 (1.6)**
Inflammatory myopathy	0 (0)	1 (2.8)	**1 (1.6)**
Myasthenia gravis	0 (0)	1 (2.8)	**1 (1.6)**
Pemphigus vulgaris	0 (0)	1 (2.8)	**1 (1.6)**
Anti-synthetase syndrome and polymyositis	1 (4)	0 (0)	**1 (1.6)**
Dermatomyosis	0 (0)	1 (2.8)	**1 (1.6)**
Systemic sclerosis	1 (4)	0 (0)	**1 (1.6)**
Inclusion body myositis	1 (4)	0 (0)	**1 (1.6)**
Idiopathic nephrotic syndrome	1 (4)	0 (0)	**1 (1.6)**
Autoimmune hemolytic anemia	1 (4)	0 (0)	**1 (1.6)**
Good’s syndrome	1 (4)	0 (0)	**1 (1.6)**
Good’s syndrome and myasthenia gravis	0 (0)	1 (2.8)	**1 (1.6)**
Atrophying polychondritis	1 (4)	0 (0)	**1 (1.6)**
**Treatment N (%)**			
Corticosteroids	16 (64)	21 (58.3)	**37 (60.7)**
Antimetabolites N (%)	13 (52)	14 (38,9)	**27 (44.3)**
Methotrexate	5	7	**12**
Mycophenolate Mofetil	5	3	**8**
Azathioprine	3	4	**7**
Anti-TNF-α N (%)	4 (16)	11 (30.5)	**15 (24.6)**
Infliximab	2	4	**6**
Adalimumab	0	4	**4**
Etanercept	1	2	**3**
Golimumab	0	1	**1**
Unspecified molecule	1	0	**1**
Rituximab	2 (8)	4 (11.1)	**6 (9.8)**
Alemtuzumab	1 (4)	0 (0)	**1 (1.6)**
Anakinra	1 (4)	0 (0)	**1 (1.6)**
Fingolimod	0 (0)	1 (2.8)	**1 (1.6)**
Leflunomide	0 (0)	1 (2.8)	**1 (1.6)**
Natalizumab	0 (0)	1 (2.8)	**1 (1.6)**
Sirolimus	0 (0)	1 (2.8)	**1 (1.6)**
Trametinib	0 (0)	1 (2.8)	**1 (1.6)**
Ustekinumab	0 (0)	1 (2.8)	**1 (1.6)**
**Treatments at toxoplasmosis N (%)**			
One IS treatment (CS or other IS)	8 (32)	19 (52.8)	**27 (44.4)**
Corticosteroids	3	8	**11**
Other IS treatment	5	11	**16**
CS and one IS treatment	11 (44)	11 (30.5)	**22 (36)**
2 IS treatment	1 (4)	4 (11.1)	**5 (8.2)**
CS and two IS treatment	2 (8)	1 (2.8)	**3 (4.9)**
Unspecified molecule	2 (8)	0 (0)	**2 (3.3)**
CS and three IS treatment	0 (0)	1 (2.8)	**1 (1.6)**
No treatment	1 (4)	0 (0)	**1 (1.6)**

**Table 2:** Characteristics of *Toxoplasma* disease.

Presentation of patients	Current study	Literature	Total
** **	N = 25	N = 36	**N = 61**
Median age (extremes)	57 (29–79)	47 (18–86)	**53 (18–86)**
Sex ratio (F/M)	1.8	3	**2.27**
**Clinical characteristics N (%)**			
Neurological symptoms	**7 (28)**	**22 (61.1)**	**29 (47.5)**
Motor deficit/palsy/hemiparesis	2	16	**18**
Seizure	2	6	**8**
Speech disturbance	0	9	**7**
Cognitive impairment	3	3	**6**
Brain abscess	4	1	**5**
Behavioral changes	0	2	**2**
Ocular symptoms	**14 (56)**	**10 (27.8)**	**24 (39.3)**
Decreased vision	12	10	**22**
Retinochoroiditis	6	7	**13**
Uveitis/vitritis	8	3	**11**
Necrotizing retinitis	0	4	**4**
Floaters	1	2	**3**
General symptoms	**8 (32)**	**14 (38.9)**	**22 (36)**
Fever	6	13	**19**
Headache	0	7	**7**
Asthenia	2	2	**4**
Lymph nodes/Splenomegaly	1	1	**2**
Pulmonary symptoms	3 (12)	3 (8.3)	**6 (9.8)**
Dermatological signs	2 (8)	2 (5.5)	**4 (6.5)**
Join synovitis	0 (0)	1 (2.8)	**1 (1.6)**
**Toxoplasmosis clinical form N (%)**			
Cerebral	7 (28)	23 (63.9)	**30 (49.2)**
Ocular	11 (44)	8 (22.2)	**19 (31.1)**
Disseminated	4 (16)	4 (11.1)	**8 (13.1)**
Pauci-symptomatic	3 (12)	1 (2.8)	**4 (6.6)**
**Toxoplasmosis classification N (%)**			
Definite	22 (88)	26 (72.2)	**48 (78.7)**
Probable	3 (12)	10 (27.8)	**13 (21.3)**
**Toxoplasmosis type of infection N (%)**			
Reactivation	12 (48)	19 (52.8)	**31 (50.9)**
Primary infection	10 (40)	11 (30.5)	**21 (34.5)**
Not determinate	3 (12)	6 (16.7)	**9 (14.6)**
**Diagnostic methods**			
Serological test + PCR + medical imaging	12 (48)	7 (19.4)	**19 (31.1)**
Serological test + medical imaging	1 (4)	8 (22.2)	**9 (14.7)**
Serological test + histology + medical imaging	0 (0)	7 (19.4)	**7 (11.5)**
Serological test + PCR	5 (20)	0 (0)	**5 (8.2)**
PCR + medical imaging	3 (12)	2 (5.6)	**5 (8.2)**
Serological test + PCR + histology + medical imaging	1 (4)	3 (8.3)	**4 (6.6)**
Histology + medical imaging	0 (0)	4 (11.1)	**4 (6.6)**
Serological test	2 (8)	1 (2.8)	**3 (4.9)**
Serological test + histology	0 (0)	2 (5.6)	**2 (3.3)**
PCR + histology + medical imaging	1 (4)	1 (2.8)	**2 (3.3)**
Serological test + culture + PCR	0 (0)	1 (2.8)	**1 (1.6)**
**Treatment N(%)**			
Pyrimethamine-Sulfadiazine	10 (40)	16 (44.5)	**26 (42.6)**
Sulfamethoxazole-Trimethoprim	0 (0)	9 (25)	**9 (14.8)**
No treatment	3 (12)	5 (13.9)	**8 (13.1)**
Pyrimethamine-Azithromycin	7 (28)	0 (0)	**7 (11.5)**
Pyrimethamine-Clindamycin	1 (4)	3 (8.3)	**4 (6.6)**
Pyrimethamine	0 (0)	3 (8.3)	**3 (4.9)**
Not specified	2 (8)	0 (0)	**2 (3.3)**
Pyrimethamine-Sulfadiazine-Azithromycin	1 (4)	0 (0)	**1 (1.6)**
Azithromycin	1 (4)	0 (0)	**1 (1.6)**
**Outcome N(%)**			
Recovery	9 (36)	22 (61.1)	**31 (50.8)**
Partial recovery	9 (36)	4 (11.1)	**13 (21.3)**
Died	2 (8)	7 (19.5)	**9 (14.8)**
Not specified	5 (20)	3 (8.3)	**8 (13.1)**

Primary infections and reactivations were found in a similar proportion, and three cases were not classified because the diagnosis was made by PCR without serology. Ocular toxoplasmosis was the most frequent form of toxoplasmosis Among disseminated toxoplasmosis cases, two had pulmonary involvement: one associated with a maculo-papular rash with *T*. *gondii*-positive PCR in dermal biopsies, and the other in a patient presented a very severe clinical picture leading to a fatal cardiogenic shock. Unfortunately, information regarding the IS treatment could not be retrieved in this case. The other two cases of disseminated toxoplasmosis associated ocular and cerebral injuries.

Three pauci-symptomatic cases of toxoplasmosis presenting unusual clinical or outcome aspects were included. One case concerned a 49-year-old woman with an untreated systemic sclerosis suffering from unusual diffuse polyadenopathies associated with asthenia and myalgia. It was a primary infection, like the case of a 44-year-old woman suffering from IBD and presenting a high fever. The particularity of this last case relies in parasitic DNA detection in the blood and in the cerebrospinal fluid (CSF), without any neurological signs. Another unusual case was a reactivation of a chronic toxoplasmosis in a pregnant woman with IBD, herself presenting only fever at delivery, but leading to a congenital toxoplasmosis with fever and hepatosplenomegaly in the newborn [10].

For the diagnosis, serological test, molecular diagnosis, and medical imaging in association were largely used (48%), followed by the association of serology and PCR (20%). Histology was rarely reported as an isolated diagnostic tool in this series (2 cases) and serological tests were used without other methods in two cases (8%).

Outcome of the infection is not known for five cases (20%). Two patients died (8%) after disseminated and cerebral toxoplasmosis: death occurred very quickly, they were not able to benefit from any specific treatment for toxoplasmosis. Most patients received the classic combinations of molecules for the treatment of toxoplasmosis. A few cases of ocular toxoplasmosis or special cases have been the subject of other associations.

Microsatellite genotyping was applied to the 19 strains or DNA extract received at the NRCT. Twelve of these samples were fully genotyped: seven cases were due to a type II strain, three to atypical unclassified strains, one to a haplogroup 16 strain, and one to an Africa 4 genotype (S1 Data). The genotype could not be determined for seven strains due to an insufficient DNA amount in the extract.

### Literature review

We identified 36 clinical cases corresponding to our inclusion criteria between 1980 and 2020 (Tables 1 and 2 and S1 Data [11–44]).

The AIDs, the IS treatments most frequently associated with toxoplasmosis, and the clinical forms of toxoplasmosis are similar to those observed in our study. The proportion of cerebral form is higher compared to our series (63.9% vs 28%) and the ocular forms is lower (22.2% vs 44%).

For the diagnosis, the most used combination of technique is serology, molecular biology, and medical imaging (22.2%), but many other combinations of techniques have been used (on one case, culture was used for the diagnosis). More deaths have occurred in the literature (19.5%, only cerebral forms) and among them; four patients did not receive any treatment for toxoplasmosis because they died shortly from organic failure after or sometimes before the diagnosis of toxoplasmosis. Majority of cases were treated with classical agents, pyrimethamine-sulfadiazine (44.5%) and sulfamethoxazole-trimethoprim (25%), but in case of contraindication, other therapies like pyrimethamine alone or associated with clindamycin were also used (8.3% each).

### Statistical analysis

There were significantly more reactivations associated with cerebral toxoplasmosis cases (*p* = 0.023) and more primary-infections in disseminated forms (*p* = 0.049) (Table 3). For the pauci-symptomatic and ocular toxoplasmosis, there was no statistical difference between reactivation and primary-infection, but the analysis for pauci-symptomatic cases was done on a few numbers of cases. Due to the diversity of individual IS treatments, we chose to consider only the most used classes of molecules for comparing their impact on the clinical forms of toxoplasmosis, ocular or systemic. There was no more ocular toxoplasmosis compared to other clinical forms whether the patient treatments comprised corticosteroids, anti-TNF-α or antimetabolites, alone or associated (Table 3).

**Table 3:** Statistical analysis of the type of *Toxoplasma* infection according to clinical form and IS treatment.

	**Toxoplasmosis reactivation**	**Toxoplasmosis primary infection**	***p*-value**
Number of cases N	31	21	
Ocular toxoplasmosis N (%)	10 (58.8)	7 (41.2)	1.000
Cerebral toxoplasmosis N (%)	18 (78.3)	5 (21.7)	0.023
Disseminated toxoplasmosis N (%)	2 (25.0)	6 (75.0)	0.049
Pauci-symptomatic toxoplasmosis N (%)	1 (25.0)	3 (75.0)	0.291
	**Ocular toxoplasmosis**	**Other types of toxoplasmosis**	***p*-value**
Number of cases N	19	42	
Age (in years, median [IQR])	56 [41.50, 71.50]	50 [36, 66]	0.276
Corticosteroids N (%)	11 (29.7)	26 (70.3)	0.784
Anti-TNFα N (%)	5 (62.5)	3 (37.5)	0.094
Antimetabolites N (%)	7 (30.4)	16 (69.6)	1.000

## Discussion

This current study is the largest series reporting toxoplasmosis cases in patients with an AID and IS agents, with 61 cases from a collection of French cases over 14 years and a review of the literature over four decades. The different periods of recruitment between the literature review (1980–2020) and the collection of NRCT cases (2006–2020) has little impact as only six cases in the literature review occurred before 2006. Even if we suppose that these 61 cases are likely underestimated due to probable underreporting, we can notice that they remain rather rare, representing about 3.4% of cases of toxoplasmosis in immunocompromised patients recovered by the French NRCT (19/560). This last percentage may not reflect the reality, as data recovered via NRCT is a non-exhaustive collection of cases in immunocompromised patients with a positive *Toxoplasma* PCR.

Most of our cases (64%) are female patients, and they represent 70% of all reported cases, in accordance with AID epidemiology, for which 78% are female [45]. Among the wide variety of AIDs listed, two pathologies are clearly predominant: RA (28%) and SLE (23%), corresponding to the most common AID found in the general population. Overall, corticosteroids (alone or in combination with other SI treatments), antimetabolites and anti-TNFα are the most commonly used molecules. Although our series is the largest one described in the literature, it presented the usual limitations of a descriptive retrospective study. Without control population, it does not allow conclusion about incidence and risk factors of toxoplasmosis in these AID patients treated by immunosuppressive therapies. Multiplicity of IS treatments and their associations, the lack of data on corticosteroid doses, and the wide variety of AIDs are limits to the analysis of the data.

Regarding the clinical type, cerebral toxoplasmosis was the most frequent, and disseminated toxoplasmosis are the rarest forms (pauci-symptomatic toxoplasmosis excluded). For the whole of our cases, as in transplant recipients or HIV seropositive patients, toxoplasmosis is also most often due to reactivation of a chronic infection (50.9% reactivation versus 34.5% primary infection). Cerebral toxoplasmosis is significantly more often related to reactivation and disseminated toxoplasmosis to primary-infection.

In immunosuppressed patients, diagnosis of toxoplasmosis may be challenging, and questions about the best management strategy are still a matter of debate. We were able to see through our cases that the use of molecular biology alone makes it possible to establish with certainty the diagnosis of toxoplasmosis but cannot differentiate primary- infection from reactivation. Despite the major advances allowed by these techniques, as highlighted in the article by Dupont *et al*, serology remains an essential complement not to be neglected to make diagnosis of toxoplasmosis [46]. Facing a non-specific clinical presentation of toxoplasmosis, the major challenge is early diagnosis to increase the chances of survival. Association of serology and molecular biology can help in diagnosis of reactivation in case of increased IgG, even if we must keep in mind that i) a negative PCR does not definitively rule out a reactivation, and ii) an IgG increase is very much not specifically associated to a clinical reactivation. IgA analysis in this context allows a better characterization of toxoplasma reactivation.

The higher frequency of ocular toxoplasmosis cases in our series is probably explained by the recruitment via the French NRCT. The inclusion of ocular forms in this study may be questionable as ocular toxoplasmosis is not exclusive to immunocompromised patients. Immunocompetent patients may also develop an ocular involvement during acquired toxoplasmosis with frequency varying according to country [47]. In France, as there is no data on the incidence of ocular injury during a *Toxoplasma* infection in the immunocompetent population, it is difficult to conclude that the underlying disease or immunosuppressive therapies favored the ocular involvement. The three most used types of treatment, corticosteroids, anti-TNF-α and antimetabolites, whether alone or associated, do not promote ocular toxoplasmosis more than other toxoplasmosis forms (Table 3). However, the administration of systemic corticosteroids, without the concurrent use of antiparasitic agents, is known to lead to more severe ocular lesion [48] or to favor reactivation of *Toxoplasma* retinochoroiditis [49, 50]. Another factor influencing the development and the outcome of ocular toxoplasmosis is the parasitic strain. Notably, some strains from South America are responsible for more frequent and more severe cases of ocular toxoplasmosis due to a different local immunopathology [47,51,52]. Here, among the four genotypes characterized for the 11 cases of our ocular toxoplasmosis series, three genotypes designated as atypical were similar to genotypes circulating in South America, and one belonged to the Haplogroup 16 genotype, already described in severe cases with ocular involvement [53, 54]. What is the respective part of the strain and of immunosuppression in the occurrence of these ocular forms in AID patients? Additional data are required for a definitive answer. However, given the potential higher eye pathogenicity of these South American genotypes, European and North American physicians should inform their AID patients, seronegative for *Toxoplasma*, about the risk associated with a travel outside of these continents or eating uncooked food imported from South America. Here, two out of the three atypical strains were acquired through consumption of raw horsemeat that is generally imported fresh from South America and has already been reported as the source of severe cases of toxoplasmosis [55]. For the last atypical strain, no epidemiological risk factors were mentioned.

At the opposite of ocular cases, the systemic forms of toxoplasmosis in our series were due to type II strains in seven cases (six isolated cerebral localizations and one disseminated form), and to an Africa 4 genotype in one case (cerebral form). The type II lineage is largely predominant in European as well as in North American countries [53, 56]. Currently, the genetic population of *Toxoplasma* strains circulating in France is nearly exclusively represented by this clonal type II lineage, in animals as well as in humans [57,58]. For instance, it is observed in 95% of congenital toxoplasmosis cases in France [58]. It has also been shown to predominate in immunocompromised patients who acquired their infection in Europe whereas other genotypes were found in patients who acquired infection on the African continent or in the French West Indies [59]. Here, the Africa 4 genotype was recovered from a patient who traveled to West Africa, India, and Mauritius. Ajzenberg *et al*. noted that the distribution of genotypes (type II *vs*. non-type II) was not significantly different when patients were stratified by underlying cause of immunosuppression, site of infection, or outcome [59]. The authors concluded that in these immunocompromised patients (HIV-infected patients and haematological malignancies), immune deficiency is much more involved than parasite factors in toxoplasmosis outcome. As shown by the predominance of Type II in our French patients and an Africa genotype in an African patient, this conclusion may probably be extended to AID patients developing cerebral or other systemic forms of toxoplasmosis.

## Conclusion

The wide variety of AIDs and treatments precluded the analysis of risk factors. Because most cases were observed in patients with RA or SLE, it will be interesting to focus on these two AIDs, by conducting a large prospective study on the prevalence of *Toxoplasma* disease and identification of specific risk factors, possibly antimetabolite conventional treatments (methotrexate, azathioprine or MMF) or biologics (like anti-TNF-α). At present, the occurrence of toxoplasmosis in these situations is not well understood, and it is very difficult to highlight a link between *Toxoplasma* disease and the use of precise immunosuppressive molecule.

In this context, with the increased use of biotherapies in the treatment of AIDs, clinicians should be aware of the occurrence of toxoplasmosis at treatment onset. The frequency of this event is too low to suggest a systematic prophylaxis as in other immune deficiency. However, it seems essential to know the immune status toward *Toxoplasma* in these patients before starting treatment, and in case of occurrence of non-specific symptoms, to look for this pathology by combining molecular biology and serology with IgG, IgM and IgA analyses.

## Supporting information

S1 Data Summary of cases included in the current study. (DOCX)
