# Supplementary material for: Toxoplasmosis in patients with an autoimmune disease and immunosuppressive agents: A multicenter study and literature review
Source: PLoS Negl Trop Dis. 2022 Aug 8;16(8):e0010691. doi: 10.1371/journal.pntd.0010691 (PMC9387931; doi:10.1371/journal.pntd.0010691)
Supplement: S1 Data — (DOCX) [file pntd.0010691.s001.docx]

**Supplementary data:** summary of cases included in the current study

|  |  |  | **Auto-immune disease** | | | **Toxoplasmosis** | | | | | |  | |  | |  | |
| --- | --- | --- | --- | --- | --- | --- | --- | --- | --- | --- | --- | --- | --- | --- | --- | --- | --- |
| **Case number**  **(NRCT code)** | **Sex** | **Age** | **Type** | **Treatment** | **Disease in flare or Remission** | **Classification** | **Type** | **Primo-infection or reactivation** | **Clinical signs** | **Diagnostic method** | **Treatment** | **Genotype*** | **Outcome** | | **Ref** | |  |
| 1  (TgH33029) | M | 72 | Rheumatoid arthritis | CS Etanercept | Remission | Definite | Ocular | Primo infection | Bilateral uveitis Bilateral retinitis extensive | Serological test Positive PCR in AH | Pyrimethamine Azythromycin | ND | ND | | Current study | |  |
| 2  (TgH23049) | M | 69 | Rheumatoid arthritis | CS Anakinra Methotrexate | Remission | Definite | Ocular | Reactivation | Decreased vision Bilateral uveitis Chorioretinitis | Serological test Positive PCR in AH | Pyrimethamine Sulfadiazine | Atypical HG16 | Recovery | | Current study | |  |
| 3  (TgH24043) | M | 55 | Rheumatoid arthritis | CS Anti-TNF alpha | ND | Definite | Cerebral | Reactivation | Brain abscesses, seizure Visions disorders | Serological test Positive PCR in brain abscesses Brain imaging | Pyrimethamine Sulfadiazine | II | Partial recovery (cognitive sequelae) | | Current study | |  |
| 4  (TgH22047) | F | 77 | Rheumatoid arthritis | Methotrexate | Remission | Definite | Ocular | ND | Decreased vision Chorioretinitis | Positive PCR in AH | Azithromycin | Atypical | Partial recovery (loss of vision) | | Current study | |  |
| 5  (TgH30024) | F | 71 | Rheumatoid arthritis | CS  Methotrexate | ND | Definite | Ocular | ND | Decreased vision | Positive PCR in AH | Pyrimethamine Azythromycin | Atypical | Partial recovery | | Current study | |  |
| 6  (TgH33015) | F | 71 | Systemic lupus erythematosus | CS Rituximab Alemtuzumab | Disease in flare | Definite | Ocular | Primo infection | Uveitis Decreased vision | Serological test Positive PCR in AH | Pyrimethamine Azythromycin | ND | Partial recovery | | Current study | |  |
| 7  (TgH29059) | F | 46 | Systemic lupus erythematosus | CS  MMF | Disease in flare | Definite | Cerebral | Reactivation | Confusion Cognitive impairment | Serological test Positive PCR in blood and CSF | Pyrimethamine Sulfadiazine | Africa 4 | Partial recovery (cognitive sequelae) | | Current study | |  |
| 8  (TgH29073) | F | 49 | Systemic lupus erythematosus | CS  MMF | ND | Definite | Ocular | Reactivation  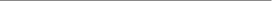Primo infection | Decreased vision Bilateral chorioretinitis | Serological test Positive PCR in blood and AH | Pyrimethamine Sulfadiazine Azithromycin | ND | Recovery | | Current study | |  |
| 9 | F | 31 | Systemic lupus erythematosus | CS Azathioprine | Disease in flare | Probable | Ocular |  | Asthenia, decreased vision Floaters, uveitis | Serological test | Pyrimethamine Azythromycin | ND | Recovery | | Current study | |  |
| 10  (TgH13117) | F | 50 | Systemic lupus erythematosus | CS Mycophenolate | Remission | Definite | Disseminated (ocular, cerebral) | Primo infection | Pan uveitis, bilateral chorioretinitis Decreased vision Maculo-papular rash, fever, cough | Serological test Positive PCR in VH/AH, Blood and CSF | Pyrimethamine Sulfadiazine | Atypical | Partial recovery (loss of vision) | | Current study | |  |
| 11 | F | 30 | Crohn disease | Azathioprine | Remission | Probable | Pauci-symptomatic | Reactivation | Fever at delivery with congenital transmission (fever and  hepatosplenomegaly in newborn) | Serological test | No | ND | Recovery | | [1] | |  |
| 12 | F | 73 | Crohn disease | Azathioprine | Remission | Definite | Ocular | Reactivation | Decreased vision Chorioretinitis | Serological test Positive PCR in AH | Pyrimethamine Azythromycin | ND | Partial recovery | | Current study | |  |
| 13  (TgH11030) | M | 66 | Crohn disease | Methotrexate | ND | Definite | Cerebral | Reactivation | Fever, confusion, cerebral abscesses Gait disturbance | Serological test Positive PCR in brain biopsy Brain imaging | Pyrimethamine Sulfadiazine | II | ND | | Current study | |  |
| 14  (TgH21084) | F | 44 | Crohn disease | Remicade  MMF | ND | Definite | Pauci-symptomatic | Primo infection | Fever | Positive PCR in blood and CSF Serological test | Pyrimethamine Clindamycin | II | Recovery | | Current study | |  |
| 15  (TgH37004) | F | 71 | Anti-synthetase syndrome Polymyositis | MMF CS | ND | Definite | Cerebral | Reactivation | Motor deficit, convulsion Brain abscesses | Serological test Positive PCR in brain biopsy and CSF Brain imaging | No | II | Death | | Current study | |  |
| 16  (TgH29066) | M | 55 | Atrophying polychondritis | ND | ND | Definite | Pulmonary | Primo infection | Pneumonia Cardiogenic shock | Serological test Positive PCR in blood and BAL | No | II | Death | | Current study | |  |
| 17 | M | 79 | Autoimmune hemolytic anemia | CS | Disease in flare | Definite | Ocular | Reactivation | Decreased vision | Serological test Positive PCR in AH | Pyrimethamine Azythromycin | ND | ND | | Current study | |  |
| 18 | M | 57 | Body myositis | CS | Remission | Definite | Ocular | Reactivation | Decreased vision Chorioretinitis, uveitis | Serological test Positive PCR in AH | Pyrimethamine Azythromycin | ND | Recovery | | Current study | |  |
| 19  (TgH31039) | F | 71 | Cryoglobulinemia type 1 | CS Rituximab | ND | Definite | Cerebral | Reactivation | Temporo-spatial disorientation Confusion, dysarthria Impairment of cognitive functions | Serological test Positive PCR in CSF Cytology of brain biopsy | Pyrimethamine Sulfadiazine | II | Partial recovery (cognitive sequelae) | | Current study | |  |
| 20  (TgH23082) | M | ND | Good's syndrome | ND | ND | Definite | Cerebral | Reactivation | Cognitive impairment,  Multiple brain abscess | Brain imaging Histology of brain biopsy Positive PCR in brain biopsy | Pyrimethamine  Sulfadiazine | II | Partial recovery | | Current study | |  |
| 21  (TgH13168) | F | 29 | Idiopathic nephrotic syndrome | CS  MMF | Remission | Definite | Disseminated (ocular, cerebral) | Primo infection | Uveitis, Decreased vision  Fever | Serological test Positive PCR in AH and CSF | Pyrimethamine Sulfadiazine | ND | Recovery | | Current study | |  |
| 22  (TgH21079) | F | 67 | Psoriatic arthritis | Methotrexate | Remission | Definite | Disseminated  (lung, skin) | Primo infection | Fever, polypnea, oxygen-dependence Maculo-papular rash | Serological test Positive PCR in BAL and skin | Pyrimethamine Sulfadiazine | ND | Recovery | | Current study | |  |
| 23  (TgH10022) | F | 75 | Psoriatic arthritis | CS  Remicade | ND | Definite | Cerebral | ND | Neurological symptoms | Positive PCR in CSF | ND | ND | ND | | Current study | |  |
| 24  (TgH33018) | M | 56 | Sarcoidosis | CS | Remission | Definite | Ocular | Primo infection | Decreased vision Uveitis | Serological test Positive PCR in AH | Pyrimethamine Azythromycin | ND | Partial recovery (retinal detachment) | | Current study | |  |
| 25 | F | 49 | Scleroderma | No | Remission | Probable | Pauci-symptomatic | Primo infection | Asthenia, myalgia,  Polyadenopathies cervical, axillary and inguinal | Serological test | Pyrimethamine Sulfadiazine | ND | Recovery | | Current study | |  |
| 26 | F | 64 | Rheumatoid arthritis | CS Methotrexate Adalimumab | Disease in flare | Definite | Cerebral | Reactivation | Seizure | Serological test Positive PCR in brain biopsy Brain imaging | Sulfamethoxazole  Trimethoprim | NA | ND | | [2] | |  |
| 27 | M | 67 | Rheumatoid arthritis | Adalimumab | Remission | Definite | Cerebral | ND | Headaches, gait and speech disturbances  Facial palsy and mild hemiparesis | Positive PCR in brain biopsy Brain imaging | Pyrimethamine Sulfadiazine | NA | ND | | [3] | |  |
| 28 | F | 76 | Rheumatoid arthritis | Methotrexate Infliximab | ND | Definite | Cerebral | Reactivation | Right facial droop Slurred speech and difficulty walking | Serological test Histology, brain imaging | Pyrimethamine Clindamycin | NA | Recovery | | [4) | |  |
| 29 | M | 43 | Rheumatoid arthritis | Methotrexate Infliximab | ND | Definite | Ocular | Primo infection | Decreased vision Chorioretinitis | Serological test Positive PCR in AH | Pyrimethamine Sulfadiazine | NA | Recovery | | [5] | |  |
| 30 | F | 40 | Rheumatoid arthritis | Etanercept | Remission | Probable | Ocular | Reactivation | Decreased vision, peripapillary edema,  inflammation | Serological test Fundus | Pyrimethamine Sulfadiazine | NA | Recovery | | [5] | |  |
| 31 | F | 36 | Rheumatoid arthritis | CS, Methotrexate Infliximab Leflunomide | ND | Definite | Cerebral | Reactivation | Headache, seizure, speech disturbance,  Facial hemiparesis | Serological test Histology, brain imaging | Pyrimethamine | NA | Recovery | | [6] | |  |
| 32 | F | 55 | Rheumatoid arthritis | CS Methotrexate | Remission | Definite | Disseminated (lungs) | Reactivation | Non-productive cough, shortness of breath | Serological test Histology | Sulfamethoxazole  Trimethoprim | NA | Recovery | | [7] | |  |
| 33 | F | 65 | Rheumatoid arthritis | Methotrexate | Remission | Definite | Cerebral | Reactivation | Sensory hypoesthesia on the right face side | Serological test Positive PCR in blood and CSF Brain imaging | Sulfamethoxazole  Trimethoprim | NA | Recovery | | [8] | |  |
| 34 | F | 39 | Rheumatoid arthritis | CS | Remission | Definite | Disseminated (skin, lungs) | Reactivation | Cough, red skin macules,  Synovitis in the right knee Partial seizure | Serological test Culture Positive PCR in BAL | Pyrimethamine Sulfadiazine | NA | Recovery | | [9] | |  |
| 35 | F | 86 | Rheumatoid arthritis | Adalimumab | Remission | Definite | Ocular | Primo infection | Floaters, and decreased vision Uveitis, necrotizing retinitis | Serological test Positive PCR in AH | Sulfamethoxazole  Trimethoprim | NA | Recovery | | [10] | |  |
| 36 | M | 65 | Rheumatoid arthritis | Trametinib | ND | Definite | Disseminated (ocular, cerebral) | Primo infection | Gait incoordination and fine motor skill difficulties | Serological test Positive PCR in CSF Brain imaging | Sulfamethoxazole  Trimethoprim | NA | Recovery | | [11] | |  |
| 37 | F | 70 | Rheumatoid arthritis  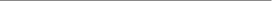  Systemic lupus erythematosus | Infliximab Methotrexate | ND | Definite | Cerebral | Reactivation | Right sided weakness Mild left-sided facial droop and slurred speech | Brain histology and imaging Serological test | Sulfamethoxazole  Trimethoprim | NA | ND | | [12] | |  |
| 38 | F | 43 |  | CS Azathioprine | Disease in flare | Definite | Disseminated (ocular, cerebral) | Primo infection | Fever, macular rash, decreased vision Retinochoroiditis | Serological test Positive PCR in CSF Histology, brain imaging | Pyrimethamine Sulfadiazine | NA | Recovery | | [13] | |  |
| 39 | F | 36 | Systemic lupus erythematosus | CS Sirolimus | ND | Definite | Cerebral | ND | Pneumonia (no neurological signs) | Histology, brain imaging | No | NA | Death | | [14] | |  |
| 40 | M | 18 | Systemic lupus erythematosus | CS | Disease in flare | Probable | Cerebral | Reactivation | Fever, seizure, speech disturbance | Serological test Brain imaging | Pyrimethamine Clindamycin | NA | Recovery | | [15] | |  |
| 41 | F | 47 | Systemic lupus erythematosus | CS | Disease in flare | Definite | Ocular | Reactivation | Decreased vision, necrotizing retinitis, seizure | Serological test Positive PCR in AH Histology | Pyrimethamine Sulfadiazine | NA | Partial recovery (loss of vision) | | [16] | |  |
| 42 | F | 24 | Systemic lupus erythematosus | CS | Remission | Definite | Cerebral | Reactivation | Decreased vision, transient unconsciousness Hemiparesis | Serological test Histology, brain imaging | Pyrimethamine Sulfadiazine | NA | Recovery | | [17] | |  |
| 43 | F | 48 | Systemic lupus erythematosus | CS | Disease in flare | Definite | Cerebral | Reactivation | Seizure, confusion | Histology | No | NA | Death | | [18] | |  |
| 44 | M | 47 | Systemic lupus erythematosus | CS | ND | Definite | Cerebral | Reactivation | Speech disturbance, cognitive impairment Wide-base gait | Serological test Histology, brain imaging | Sulfamethoxazole  Trimethoprim | NA | Recovery | | [19] | |  |
| 45 | F | 44 | Systemic lupus erythematosus | CS | ND | Definite | Cerebral | ND | Fever, headache, confusion | Histology, brain imaging | No | NA | Death | | [20] | |  |
| 46 | F | 50 | Systemic lupus erythematosus | CS | ND | Definite | Cerebral | Primo infection | Fever, palsy, cranial neuropathy, hemiparesis, ataxia | Serological test Histology, brain imaging | No | NA | Death | | [21] | |  |
| 47 | F | 71 | Cutaneous vasculitis  Type I Cryoglobulinemia | CS Rituximab | Remission | Definite | Cerebral | Reactivation | Speech disturbance Behavioral changes | Serological test Positive PCR in CSF Histology, brain imaging | Pyrimethamine Sulfadiazine | NA | Partial recovery (cognitive sequelae) | | [22] | |  |
| 48 | F | 25 | Ankylosing Spondylitis | Etanercept | ND | Probable | Ocular | Reactivation | Decreased vision, chorioretinitis | Serological test Fundus | Sulfamethoxazole  Trimethoprim | NA | Recovery | | [23] | |  |
| 49 | F | 48 | Connective tissue disease | CS MMF | ND | Definite | Cerebral | Reactivation | Fever, confusion, hemiparesis | Serological test Histology, brain imaging | Pyrimethamine Sulfadiazine | NA | Death | | [24] | |  |
| 50 | F | 22 | Dermatomyositis | Azathioprine Rituximab | Remission | Definite | Cerebral | ND | Dysarthria, central facial paralysis, headache, vomit | Positive PCR in CSF | Pyrimethamine Sulfadiazine | NA | Partial recovery (cognitive sequelae) | | [25] | |  |
| 51 | F | 33 | inflammatory bowel disease | CS Azathioprine | Remission | Probable | Cerebral | Reactivation | Headaches, fever, vomiting | Serological test Histology | Pyrimethamine | NA | Recovery | | [26] | |  |
| 52 | F | 33 | inflammatory bowel disease | Azathioprine | ND | Probable | Ocular | Reactivation | Decreased vision, peri-ocular pain | Serological test Fundus | Pyrimethamine Sulfadiazine | NA | Recovery | | [27] | |  |
| 53 | F | 47 | inflammatory bowel disease | CS Adalimumab | ND | Probable | Ocular | Primo infection | Decreased vision,  Necrotizing retinochoroiditis | Serological test Fundus | Pyrimethamine Sulfadiazine | NA | Recovery | | [28] | |  |
| 54 | F | 72 | Inflammatory myopathy | Rituximab | Remission | Definite | Cerebral | Primo infection | Fever, confusion Generalized weakness. | Serological test Positive PCR in CSF Brain imaging | Pyrimethamine Sulfadiazine | NA | Death | | [29] | |  |
| 55 | M | 28 | Multiple sclerosis | Natalizumab | Remission | Probable | Ocular | Reactivation | Floaters, decreased vision, Iritis, chorioretinitis | Serological test Fundus | Pyrimethamine Sulfadiazine | NA | Recovery | | [30] | |  |
| 56 | F | 30 | Multiple sclerosis | CS Fingolimod | Remission | Probable | Cerebral | Primo infection | Asthenia, fever, hemiparesis | Serological test Brain imaging | Pyrimethamine Clindamycin | NA | Recovery | | [31] | |  |
| 57 | M | 77 | Myasthenia gravis | CS MMF | Remission | Definite | Cerebral | Primo infection | Weakness, speech, and gait difficulty Cognitive impairment | Serological test Positive PCR in CSF Brain imaging | Pyrimethamine Sulfadiazine | NA | Death | | [29] | |  |
| 58 | F | 54 | Myasthenia gravis Good’s Syndrome | CS MMF | Remission | Definite | Cerebral | ND | Headache, facial weakness  Decreased visual acuity | Positive PCR in brain biopsy Histology, brain imaging | Pyrimethamine Sulfadiazine | NA | Recovery | | [32] | |  |
| 59 | F | 52 | Pemphigus vulgaris | Rituximab | Remission | Probable | Cerebral | Primo infection | Persistent rhinorrhea and neurological symptoms | Serological test Brain imaging | Pyrimethamine | NA | Recovery | | [33] | |  |
| 60 | M | 26 | Psoriasis arthritis | Ustekinumab | ND | Probable | Pauci-symptomatic | Primo infection | Asthenia, fever, sweating, weight loss Bilateral occipital, cervical, axillary and inguinal lymph adenomegalies, splenomegaly | Serological test | No | NA | Recovery | | [34] | |  |
| 61 | M | 25 | Sarcoidosis | CS Golimumab | ND | Definite | Cerebral | ND | Headache, vomiting, decreased vision Facial palsy | Histology, brain imaging | Sulfamethoxazole  Trimethoprim | NA | Partial recovery (cognitive sequelae) | | [35] | |  |

* Genotyping results of 15 microsatellite markers in a single multiplex PCR assay;
F: female; M: male; MMF: mycophenolate mofetil; CS: corticosteroids; CSF: cerebrospinal fluid; PCR: Polymerase Chain Reaction; ND: Not determined; NA: non amplified; AH: aqueous humor; VH: vitreal humor

References:

1. Bonnet Ducrot S, Plantaz D, Mathieu N, Debillon T, Bost Bru C, Brenier-Pinchart M-P, et al. Neonatal fever: A puzzling case. Arch Pediatr. 2018;25(7):435–8.

2. Cren J-B, Bouvard B, Crochette N. Cerebral toxoplasmosis and anti-TNFα: a case report. IDCases. 2016;5:40.

3. Nardone R, Zuccoli G, Brigo F, Trinka E, Golaszewski S. Cerebral toxoplasmosis following adalimumab treatment in rheumatoid arthritis. Rheumatology. 2014;53(2):284–284.

4. Pulivarthi S, Reshi RA, McGary CT, Gurram MK. Cerebral toxoplasmosis in a patient on methotrexate and infliximab for rheumatoid arthritis. Intern Med. 2015;54(11):1433–6.

5. Lassoued S, Zabraniecki L, Marin F, Billey T. Toxoplasmic chorioretinitis and antitumor necrosis factor treatment in rheumatoid arthritis. Semin Arthritis Rheum. 2007;36(4):262–3.

6. Young JD, McGwire BS. Infliximab and reactivation of cerebral toxoplasmosis. N Engl J Med. 2005 Oct 6;353(14):1530–1; discussion 1530-1531.

7. Abdulkareem A, D’Souza RS, Patel N, Donato AA. A rare case of pulmonary toxoplasmosis in a patient with undifferentiated inflammatory arthritis on chronic methotrexate and corticosteroid therapy. BMJ Case Rep. 2017

8. Matsuura J, Fujii A, Mizuta I, Norose K, Mizuno T. Cerebral toxoplasmosis diagnosed by nested-polymerase chain reaction in a patient with rheumatoid arthritis. Intern Med. 2018;57(10):1463–8.

9. Vittecoq O, Mejjad O, Voisin L, Silva FD, Dominique S, Jouen-Beades F, et al. Septic arthritis of the knee with Toxoplasma gondii in a patient with rheumatoid arthritis. J Clin Rheumatol. 1995;1(5):299–301.

10. Walkden A, Wig S, Bhatt PR, Jones NP, Steeples LR. Atypical ocular toxoplasmosis during adalimumab anti–tumor necrosis factor therapy for rheumatoid arthritis: J Clin Rheumatol. 2019;1.

11. Gharamti AA, Rao A, Pecen PE, Henao-Martínez AF, Franco-Paredes C, Montoya JG. Acute toxoplasma dissemination with encephalitis in the era of biological therapies. Open Forum Infect Dis. 2018;5(11):ofy259.

12. Hill B, Wyatt N, Ennis D. Cerebral toxoplasmosis in a rheumatoid arthritis patient on immunosuppressive therapy. Cureus. 2020;12(6):e8547.

13. Furuya H, Ikeda K, Iida K, Suzuki K, Furuta S, Tamachi T, et al. Disseminated toxoplasmosis with atypical symptoms which developed with exacerbation of systemic lupus erythematosus. Lupus. 2019;28(1):133–6.

14. Murro D, Novo J, Arvanitis L. Asymptomatic diffuse “encephalitic” cerebral toxoplasmosis in a woman with systemic lupus erythematosus. J Clin Neurosci. 2016;29:194–6.

15. Pagalavan L, Kan FK. Cerebral toxoplasmosis in systemic lupus erythematosus following intravenous methylprednisolone. Med J Malaysia. 2011;66(1):68–70.

16. Yamamoto JH. Severe bilateral necrotising retinitis caused by Toxoplasma gondii in a patient with systemic lupus erythematosus and diabetes mellitus. Br J Ophthalmol. 2003;87(5):651–2.

17. Seta N, Shimizu T, Nawata M, Wada R, Mori K, Sekigawa I, et al. A possible novel mechanism of opportunistic infection in systemic lupus erythematosus, based on a case of toxoplasmic encephalopathy. Rheumatology. 2002;41(9):1072–3.

18. Zamir D, Amar M, Groisman G, Weiner P. Toxoplasma infection in systemic lupus erythematosus mimicking lupus cerebritis. Mayo Clin Proc. 1999;74(6):575–8.

19. Huang Y, Lieu A, Chen Y-T. A rare presentation of toxoplasma encephalitis in systemic lupus erythematous patient: case report and review of literature. Int J Adv Med. 2015;414–8.

20. Lummus S, Kleinschmidt-DeMasters BK. Predominantly periventricular necrotizing encephalitis due to toxoplasmosis: two unusual cases and review of literature. Clin Neuropathol. 2014;33(1):29–37.

21. Wilson WB, Sharpe JA, Deck JHN. Cerebral blindness and oculomotor nerve palsies in toxoplasmosis. Am J Ophthalmol. 1980;89(5):714–8.

22. Safa G, Darrieux L. Cerebral Toxoplasmosis after Rituximab Therapy. JAMA Intern Med. 2013;173(10):924–6.

23. Batmaz I, Turkçu F. Toxoplasma chorioretinitis subsequent to anti-tumour necrosis factor alpha treatment in a patient with ankylosing spondylitis. West Indian Med J. 2015;64(2):168–9.

24. Pistacchi M, Gioulis M, Zirillo M, Francavilla E, Zambito Marsala S. Cerebral toxoplasmosis in undifferentiated connective disease treated with mycophenolate mofetil: an unusual case report. Acta Neurol Belg. 2016;116(4):633–6.

25. Castaño‐Amores C, Nieto‐Gómez P. Cerebral toxoplasmosis associated with treatment with rituximab, azathioprine and prednisone for dermatomyositis. Br J Clin Pharmacol. 2021;87(3):1525–8.

26. Assimakopoulos SF, Stamouli V, Dimitropoulou D, Spiliopoulou A, Panos G, Anastassiou ED, et al. Toxoplasma gondii meningoencephalitis without cerebral MRI findings in a patient with ulcerative colitis under immunosuppressive treatment. Infection. 2015;43(5):589–93.

27. Puga M, Carpio D, Sampil M, Zamora MJ, Fernandez-Salgado E. Ocular toxoplasmosis reactivation in a patient with inflammatory bowel disease under treatment with azathioprine: J Clin Gastroenterol. 2016;50(7):610.

28. Radwan A, Baheti U, Arcinue CA, Hinkle DM. Acute unilateral toxoplasma retinochoroiditis associated with adalimumab, a tumor necrosis factor-α antagonist. Retin Cases Brief Rep. 2013;7(2):152–4.

29. Bernardo DR, Chahin N. Toxoplasmic encephalitis during mycophenolate mofetil immunotherapy of neuromuscular disease. Neurol Neuroimmunol Neuroinflamm. 2015;2(1).

30. Zecca C, Nessi F, Bernasconi E, Gobbi C. Ocular toxoplasmosis during natalizumab treatment. Neurology. 2009;73(17):1418–9.

31. Enriquez-Marulanda A, Valderrama-Chaparro J, Parrado L, Diego Vélez J, Maria Granados A, Luis Orozco J, et al. Cerebral toxoplasmosis in an MS patient receiving Fingolimod. Mult Scler Relat Disord. 2017;18:106–8.

32. Sasson SC, Davies S, Chan R, Davies L, Garsia R. Cerebral toxoplasmosis in a patient with myasthenia gravis and thymoma with immunodeficiency/Good’s syndrome: a case report. BMC Infect Dis. 2016;16(1).

33. Lee EB, Ayoubi N, Albayram M, Kariyawasam V, Motaparthi K. Cerebral toxoplasmosis after rituximab for pemphigus vulgaris. JAAD Case Reports. 2020;6(1):37–41.

34. Muslimani MA, Palma-Grisi JD. Severe acute toxoplasmosis infection following ustekinumab treatment in a patient with psoriasis vulgaris. BMJ Case Reports CP. 2019;12(8).

35. Misra DP, Chengappa KG, Mahadevan A, Jain VK, Negi VS. Sarcoidosis, neurotoxoplasmosis and golimumab therapy. QJM. 2016;109(12):817–8.
